# Evaluation of Resident Evacuations in Urban Rainstorm Waterlogging Disasters Based on Scenario Simulation: Daoli District (Harbin, China) as an Example

**DOI:** 10.3390/ijerph111009964

**Published:** 2014-09-26

**Authors:** Peng Chen, Jiquan Zhang, Lifeng Zhang, Yingyue Sun

**Affiliations:** 1 College of Tourism and Geography Science, Jilin Normal University, Siping, Jilin 136000, China; E-Mails: pp11290@163.com (P.C.); zhanglf@126.com (L.Z.); syy800201@126.com (Y.S.); 2 School of the Environment, Northeast Normal University, Changchun, Jilin 130000, China

**Keywords:** scenario simulation, rainstorm waterlogging disasters, evacuation difficulty levels, Daoli District

## Abstract

With the acceleration of urbanization, waterlogging has become an increasingly serious issue. Road waterlogging has a great influence on residents’ travel and traffic safety. Thus, evaluation of residents’ travel difficulties caused by rainstorm waterlogging disasters is of great significance for their travel safety and emergency shelter needs. This study investigated urban rainstorm waterlogging disasters, evaluating the impact of the evolution of such disasters’ evolution on residents’ evacuation, using Daoli District (Harbin, China) as the research demonstration area to perform empirical research using a combination of scenario simulations, questionnaires, GIS spatial technology analysis and a hydrodynamics method to establish an urban rainstorm waterlogging numerical simulation model. The results show that under the conditions of a 10-year frequency rainstorm, there are three street sections in the study area with a high difficulty index, five street sections with medium difficulty index and the index is low at other districts, while under the conditions of a 50-year frequency rainstorm, there are five street sections with a high difficulty index, nine street sections with a medium difficulty index and the other districts all have a low index. These research results can help set the foundation for further small-scale urban rainstorm waterlogging disaster scenario simulations and emergency shelter planning as well as forecasting and warning, and provide a brand-new thought and research method for research on residents’ safe travel.

## 1. Foreword

Residents’ evacuation difficulty level refers to the level of difficulty when a disaster occurs and residents are unable to complete the evacuation process constrained by events in the external environment and residents’ own characteristics and must take shelter measures. The frequency, intensity, and resulting damage caused by urban natural disasters are constantly increasing with the acceleration of global change and urbanization [1,2,3,4,5,6,7]. In the entire city residents rainstorm waterlogging disaster emergency shelter process, the difficulty of finding refuge is an important part of the study of such events, but the need to reduce the evacuation time and to improve the success rate of emergency measures are also important factors. Drabek, the earliest scholar involved in this type of study, thought that some refuge factors affecting residents’ refuge-seeking behavior are residents’ own determination, family values and so on [8]. Whyte believes that the main elements of the conscious decision to seek refuge are personal awareness of the reality of dangers and personal understanding level [9]. Japanese scholars began to refine how the residents seek refuge after disasters by studying how the refuge seeking steps after a seek refuge notification is issued obey the sequence getting the notification → understanding the notification → believing or not believing the notification → personalizing the notification → confirming the authenticity of the notification → disaster prevention response and other continuous processes to conduct a detailed study of this topic [10,11,12]. In recent work the American scholar Sorensen decribed how promoting and inhibiting factors can affect the following three major aspects of refuge seeking activity: hazard identification, the implementation of evacuation decisions and social factors. Also after the residents decide to take refuge, affecting evacuation action factors are selection of refuge tools, asylum features, and family concepts. In the late 1980s, the Japanese Institute of Civil Engineering Construction researched the residents’ evacuation after urban waterlogging disasters from a qualitative research direction to a quantitative development, and while urban waterlogging disaster evacuation action research is related to various factors, they began using quantitative methods to analyze refuge factors and quantifying features such as housing construction, water depth, water persistence time, and presence or absence of evacuation orders [13,14,15,16]. Therefore, carrying out relevant research on urban rainstorm waterlogging disaster emergency shelter has important practical significance. From the whole process of residents’ emergency shelter seeking activity in urban rainstorm waterlogging disasters, the research on evacuation difficulty level is an important link to residents’ emergency shelter and is an important measure to reduce evacuation time and improve the success rate of evacuations as well. It has been found by summarizing existing research that most scholars have studied the process of residents’ emergency shelter only from the point of the environmental factors of disasters during residents’ evacuation processes, but they have largely ignored the influencing factors arising from the residents themselves. Consequently, this paper puts forward a concept and method for evaluation of the difficulty of residents’ evacuation during urban rainstorm waterlogging disasters, creating a difficulty evaluation model and paradigm for residents’ evacuation in these disasters considering the residents’ own vulnerabilities and cognitive risk perspectives, combined with the environmental impact of waterlogging disasters, so as to enrich and improve the theory and method of small-scale residents’ evacuation after urban rainstorm waterlogging disasters and to provide a basis for formulating urban waterlogging disaster risk management and planning in our country. This research is consistent with the research trends of small-scale disaster emergency management evaluation being carried out in many big cities at home and abroad at present. This type of research as a basis for urban disaster prevention and mitigation management as well as a basis and premise for urban disaster emergency management has not yet been launched in our country.

## 2. Overview for the Research Area

### 2.1. Natural Conditions

**Figure 1:**
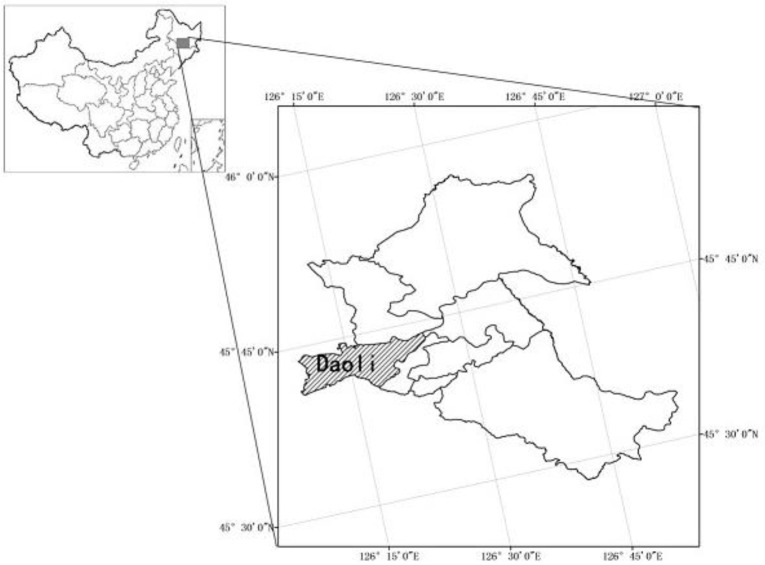
Geographical location of the study area.

Daoli District in Harbin is a part of the Songnen Plain (Figure 1). It presents as a low-lying terrain to the south and a high north area and it naturally forms flooded soil areas on both sides of the Songhua River. In recent years, over-exploitation of groundwater in this area has led to ground subsidence in some regions, where roads are therefore prone to cause the urban waterlogging disasters.

Annual precipitation on the drainage basin has been around 525 mm for many years. The occurrence of rainstorms is mostly concentrated in two months, namely July and August, where the frequency of occurrence accounts for 84%–88% of the total number of rainstorms, which indicates that July and August are the frequent rainstorm period, and rainstorm waterlogging disasters easily occur during this period. The total area of this district is 479 km^2^, with a total number of households of 179,000 and a population of some 670,000. There are 8334 households living in bungalows and 2185 households living on the first floor or in the basements of buildings. These residents living in one-story houses and below the first floor level (in basements) are key evacuation subjects since they are the most affected by waterlogging disasters.

### 2.2. Overview of Waterlogging Disasters in the Research Area

Urbanization in Harbin has been rapid in recent years. Both the new and the old city are prone to problems such as insufficient drainage pipes, and excessively small drainage pipe cross-sections. Extended service drainage pipes account for more than 30% of the total number in the drainage network, among which 27 km of drainage pipes have been in service for about 70 years, and thus are seriously aging. Most of the drainage pipes in Harbin are joint rainwater and sewage pipelines, the density of which is 5.36 km/km^2^, or about 30% below the national stipulated value. With the population in the old city continuously increasing and the gradual increase of catchment areas, the number of drainage pipelines is increasing slowly. Therefore, waterlogging in parts of many road sections can’t be discharged. Garbage amounts are also increasing and are being discharged into the pipelines, which causes pipeline blockages and large areas of road waterlogging, threatening residents’ safe travel. Due to the big fluctuations of the urban terrain in Harbin and since the rainfall in Harbin is mainly focused in July and August, with lots of severe convection weathersystems such as thunderstorms and hailstorms, urban waterlogging easily occurs in the city.

## 3. Data Collection and Processing

Among the data needed for the research is the meteorological data concerning rainfall from 1961 to 2009 of the Meteorological Bureau of Heilongjiang Province. The spatial data included high resolution Quickbird imagery (resolution: 0.61 m) and the drainage pipe network data of Daoli District. The required basic information of the city surface, including roads, residential areas, surface roughness, area and other parameters was extracted from the Quickbird image data. All the data were analyzed and extracted using the spatial analyst tool in the ARCGIS software.

## 4. Scenario Simulation of Urban Rainstorm Waterlogging

There are many numerical modeling methods applicable to urban road waterlogging, but most of them only simulate water depth and do not calculate the flow velocity and flow direction [17,18,19,20]. When a waterlogging disaster occurs, road waterlogging and flow velocity will impact residents’ walking in water, therefore, the impact of the water depth, as well as water velocity on residents’ travel should be taken into consideration. In the study, the numerical model of urban rainstorm waterlogging takes two-dimensional unsteady flow as the base framework and adopts an irregular grid to generalize land features [21]. The one-dimensional unsteady flow of the drainage network is taken into overall consideration to establish a numerical model of rainstorm waterlogging [22,23,24,25].

During the computational process, topography and terrain features were generalized into grid coordinates, such as the types of grids, elevation, roughness coefficients, area coefficient of correction, and water discharge capacity. The between-grids water exchange was calculated on the passage around the grids. For the secondary rivers at smaller spatial scales, they were generalized to special passages, and movement of waterlogging streams within these channels was calculated using the one-dimensional unsteady flow equation. The amount of water exchanges between those special channels and grids were calculated using the broad crested weir flow formulas. The two-dimensional unsteady flow equations are: 

The continuous equations:

(1) $$\frac{\partial h}{\partial t}+\frac{\partial M}{\partial x}+\frac{\partial N}{\partial y}=0$$

The momentum equation:

(2) $$\frac{\partial M}{\partial t}+\frac{\partial \left(uM\right)}{\partial x}+\frac{\partial \left(uM\right)}{\partial y}+gh\frac{\partial H}{\partial x}+\frac{gn^{2}u\sqrt{u^{2}+v^{2}}}{h^{\frac{1}{3}}}=0$$

and:

(3) $$\frac{\partial N}{\partial t}+\frac{\partial \left(uN\right)}{\partial x}+\frac{\partial \left(uN\right)}{\partial y}+gh\frac{\partial H}{\partial y}+\frac{gn^{2}v\sqrt{u^{2}+v^{2}}}{h^{\frac{1}{3}}}=0$$

where *h* is the depth of water, *H* is the water level, *q* is the source-sink term representing the effective precipitation intensity in the model, *M* and *N* are the amounts of discharge per unit width in the *x* and *y* directions, *u* and *v* are the fraction of velocity in the *x* and *y* directions, *n* is the roughness coefficient, and *g* is the gravitational acceleration, and *t* is the time of water to grid channel. The one-dimensional unsteady flow equation is:

(4) $$\frac{\partial Q}{\partial t}+\frac{\partial }{\partial l}\left(\frac{Q^{2}}{A}\right)+gA\frac{\partial H}{\partial t}=-gAS_{f}$$

where *Q* is sectional flow, *A* is calculated cross section over the water area; is friction slope; *t* is the water reaches the passage of time; is the length of the channel grid. The broad-crested weir overflow formula is:

(5) $$Q_{j}=m\sigma_{s}\sqrt{2g}H_{j}^{3/2}$$

where is the top single-wide weir flow; *m* is broad crested weir overflow coefficient; is flooded coefficient; *H_j_* is the crest level. The flow velocity calculated formula is:

(6) $$V=\sqrt{M^{2}+N^{2}}/\text{h}$$

where *V* is the flow velocity; *M* is a single-wide flow *x* direction; *N* is a single-wide flow *y* direction; *h* is the water depth.

**Figure 2:**
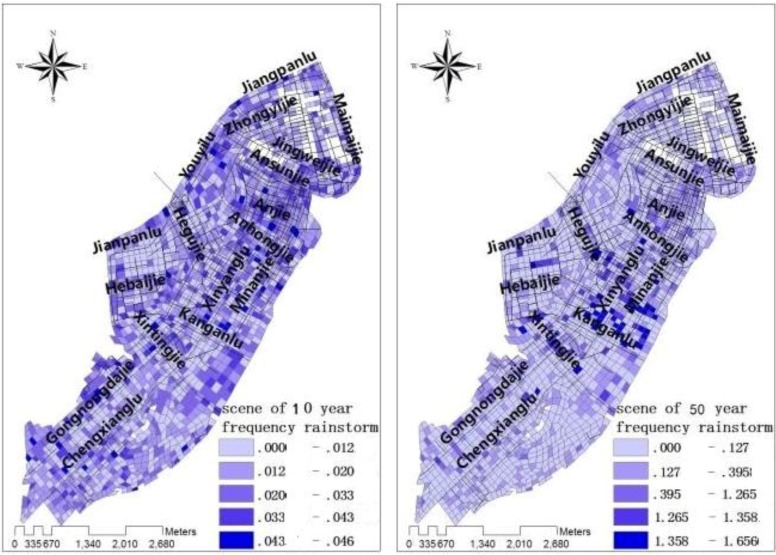
Road waterlogging status under 10-year frequency rainstorm and 50-year frequency rainstorm conditions [1].

**Figure 3:**
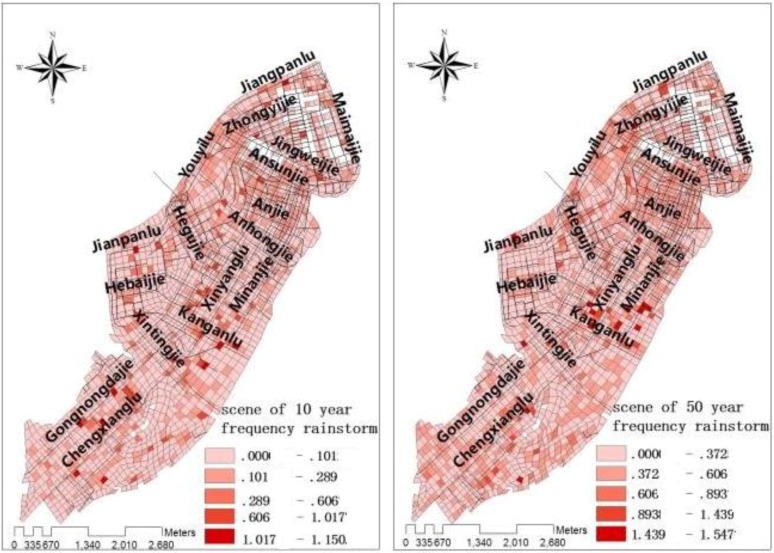
Flow velocity of road waterlogging under 10-year frequency rainstorm, and 50-year frequency rainstorm conditions [1].

Using the above model to achieve waterlogging road water and water flow speed simulation in study area, and to achieve a visual (Figure 2 and Figure 3).

## 5. Analysis of the Factors Influencing Resident Evacuation in Urban Rainstorm Waterlogging Disasters

### 5.1. Analysis of Internal Factors

**Figure 4:**
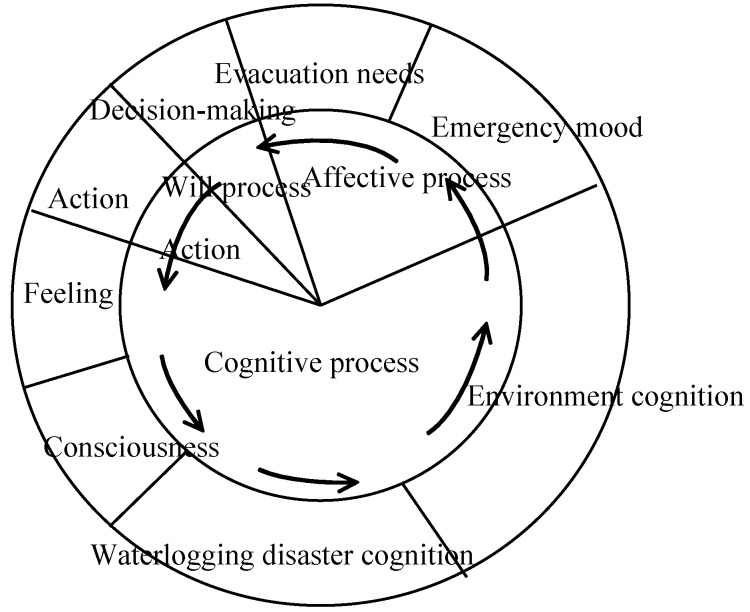
Diagram of the psychological process of evacuation behavior [26].

Residents’ evacuation refers to a series of actions and activities taken by residents when encountering emergencies or disasters. The psychology of seeking refuge is a kind of psychic reaction when residents encounter emergencies. Therefore, the psychology of residents’ evacuation directly impacts their evacuation actions, including refuge and evacuation route selection during the evacuation process. Residents’ evacuation action is the outward manifestation of the psychology of evacuation, aprocess dominated by residents’psychology (Figure 4).

#### 5.1.1. Influencing Factors and Correlation Analysis of Evacuation

Binary variables correlation analysis [26] refers to performing analysis on the degree of correlation between two or more than two variables through calculating the correlation coefficient between those variables. Based on the different types of variables being studied, it can be divided into dual distance variable correlation analysis and binary ordinal variable correlation analysis. Commonly used binary variables correlation coefficients include three types, namely Pearson simple correlation coefficient, Spearson rank correlation coefficient and Kendall rank correlation coefficient.

It has been found through the analysis of the results of the residents’ evacuation questionnaire that the content of each question is not a specific value, but an ordinal variable [27]. The value can represent a certain sequential relationship of the observed object, which is a kind of variable of “quality” factor, rather than a typical data variable. Thus, Kendall rank correlation coefficient shall be selected as the binary variables correlation coefficient to perform correlation analysis of variables [28,29,30,31].

(1) The mathematical formula of the Kendall rank correlation coefficient is:

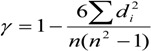
(7)
wherein, n is the sample size, *d_i_* is the level difference between each pair of two variables. The rank correlation coefficient is same as the correlation coefficient, whose value is between −1 to +1. If the value is a positive number, it indicates that two variables are positively correlated; if the value is a negative number, it indicates that two variables are negatively correlated; if the value is zero, it indicates zero correlation between two variables.

(2) The statistical formula of the Kendall rank correlation coefficient statistical test t is:

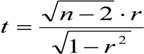
(8)
Wherein, *t* obeys the distribution of n – 2 degree of freedom. When the significance probability of *t* is *p* < 0.05,it indicates that the correlation between two variables is significant; when *p* < 0.01, it indicates that the correlation between two variables is extremely significant; when *p* > 0.05, it indicates that there is no significant correlation between two variables. The binary variable correlation analysis results of the questionnaire survey data by using SPSS software are shown in Table 1.

**Table 1:** Correlation analysis between influence factor and evacuation behavior.

Residents’ Own Characteristics	Correlation	Evacuation Shelter	Selection Tendency of Evacuation Shelter	Evacuation Method	Evacuation Route
Gender	Correlation sig. (both sides)	0.119 0.237	0.195 ** 0.036	−0.43 0.666	0.147 ** 0.032
Age	Correlation sig. (both sides)	−0.216 *** 0.010	0.152 *** 0.049	−0.063 0.448	−0.410 *** 0.002
Occupation	Correlation sig. (both sides)	−0.36 0.700	0.122 0.159	0.046 0.621	0.358 *** 0.000
Education background	Correlation sig. (both sides)	0.065 0.307	0.182 0.059	0.057 0.766	0.323 *** 0.001
Waterlogging disaster experience	Correlation sig. (both sides)	−0.154 0.126	−0.031 0.739	0.118 0.240	−0.285 *** 0.007

Notes: ** indicates that when the confidence coefficient sig (at both sides) is 0.05, two variables are significantly correlated; *** indicates that when the confidence coefficient sig. (at both sides) is 0.01, two variables are significantly correlated.

#### 5.1.2. Correlation Analysis between Residents’ Own Characteristics and Evacuation Behaviors

According to the results of the questionnaire survey, the following points concerning the evacuation behavior motivation and the evacuation behavior selection factors have been summarized through qualitative and quantitative binary variables correlation analysis:

(1) Concomitant probability values sig (at both sides) between gender factor and selection tendency of evacuation shelter and evacuation route selection are all below the significance level of 0.05, which indicates that there is a significant correlation between gender factor and selection tendency of evacuation shelter, and evacuation road selection.
(2) Concomitant probability values sig (at both sides) between age and evacuation behavior (evacuation shelter selection, and evacuation route selection) are all below the significance level of 0.01, which indicates that there is a significant linear relation between the two.
(3) Concomitant probability values sig (at both sides) between occupation and evacuation behavior (evacuation route) are all below the significance level of 0.01, which indicates that there is a significant linear relation between the two.
(4) Concomitant probability values sig (at both sides) between education background and evacuation route are below the significance level of 0.01, which indicates that there is a significant linear relation between the two.
(5) Concomitant probability values sig (at both sides) between waterlogging disaster experience and evacuation route are below the significance level of 0.05, which indicates that there is a significant linear relation between the two.

### 5.2. External Factor Analysis

During a disaster, residents’ evacuation behavior depends on the degree of influence of the waterlogging disaster and its own characteristics on the one hand, and on the other hand, the residents and the external environment and their interactions have a great influence on evacuation behavior. Residents’ evacuation behavior is formed under the combined action of external causes and internal factors. The external factors of residents’ evacuation at urban rainstorm waterlogging disasters include road grade, dangerousness of the waterlogging (waterlogging depth, flow velocity). These two aspects determine the impact of external environment factors on residents’ evacuation during the evacuation process. Road grades mainly include the provincial roads, urban main roads and township roads. Different road grades determine the traffic capacity during residents’ evacuation process. The higher the road grade, the greater the traffic capacity, and smaller on the contrary; residents’ traffic capacity and water capacity have a significant difference under different waterlogging depth and flow velocity conditions on different roads. In this section, the difficulty threshold of residents’ walking in water is determined through a residents’ walking in water experiment specified in [1].

**Figure 5:**
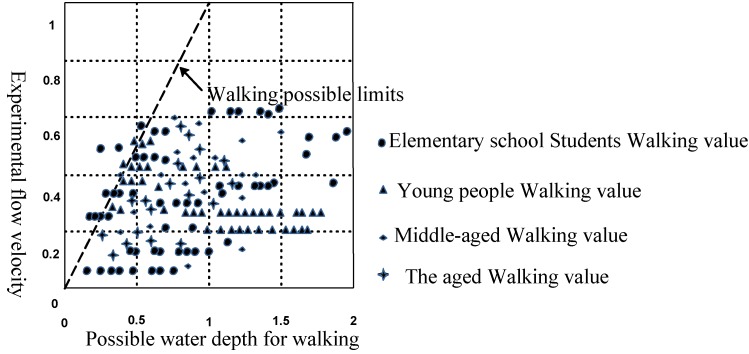
The greatest possible threshold limits for residents’ walking in water.

**Figure 6:**
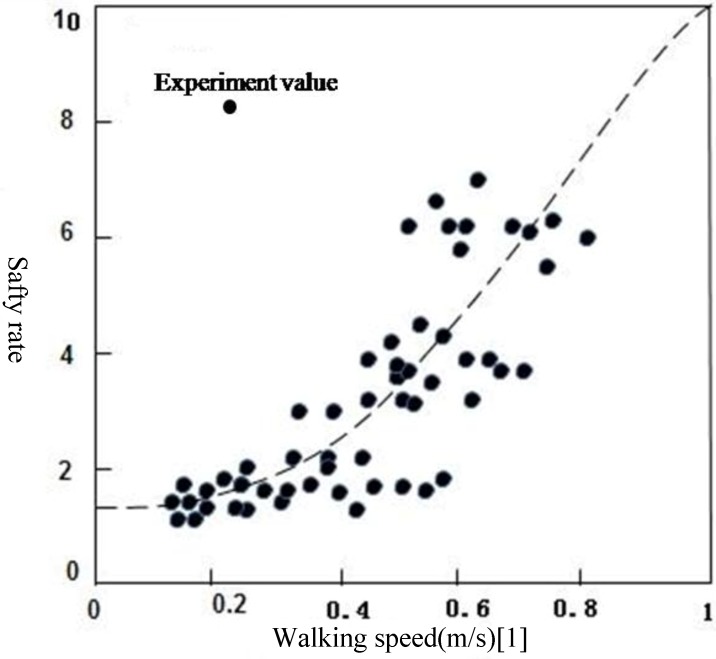
The relationship between safety rate and walking speed in evacuation process.

The greatest possible range of values of residents’ walking speed in water can be obtained by analyzing the experimental results. The greatest possible range of values of walking speed can be obtained according to the safety rate range shown in Figure 5 and Figure 6. It can be seen from the comparison between experimentally measured values and questionnaire survey results that most of the measured results of residents’ walking in water in the experiment are basically consistent with the actual questionnaire survey results. Most of the values of both results are within the range of speed with which residents can walk through water (0–1.5 m, 0–1.5 m/s).

## 6. Difficulty Evaluation Index System and Model Building for Residents’ Evacuation at Urban Rainstorm Waterlogging Disasters

### 6.1. Indicator System Selection

The difficulty evaluation indication system for residents’ evacuation at urban rainstorm waterlogging disasters is the basis of residents’ evaluation of evacuation difficulty and therefore selecting a proper indicator will or not will directly affect the quality of the overall evaluation.

**Figure 7:**
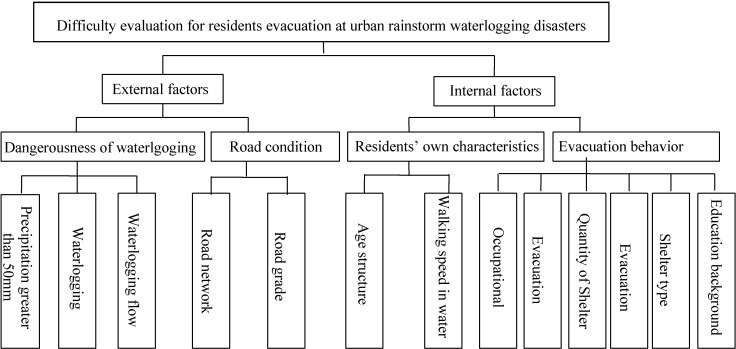
Conceptual framework for residents’ difficulty evacuation at urban rainstorm waterlogging disasters.

Therefore, in the process of indication system selection, not only the principles of the indication system construction mentioned above shall be considered, but internal factors and external factors influencing residents’ evacuation during the process of evacuation shall be taken into comprehensive consideration as well. Specifically for the above analysis results, the indication system selected is shown in Figure 7.

### 6.2. Index Quantification and Weight Calculation

#### 6.2.1. Index Quantification

Making a comprehensive survey of the qualitative evaluation indicators selected for residents’ evacuation behavior and after repeated proof, the qualitative evaluation indicators are divided into three grades according to the impact of evacuation behavior impact factor on residents’ evacuation. A score is given to each grade (Table 2). According to the difficulty evaluation concept framework for residents’ evacuation during urban rainstorm waterlogging disasters, 11 evaluation indexes were selected which have representativeness, pertinence and easily quantified features. In order to eliminate the calculation inconvenience caused by the use of different units, non-dimensionalization was applied to each index in this section:

(9) $$X_{ij}^{'}=\frac{X_{ij}}{\frac{1}{n}\sum_{i=1}^{n}X_{ij}}$$

where item *j* is the index value of the *i*th object, which is the item j index value of the *i*th object after nondimensionalization. An assignment method is adopted when there is difficulty in establishing a quantitative or qualitative index (Table 2).

#### 6.2.2. Weight Calculation

Weight valuation is needed to describe the relative degree of importance of an index in the process of evaluation, namely its weight [32]. This paper adopts the AHP method to calculate the index weight and establish the difficulty evaluation index system and weight of residents’ evacuation at urban rainstorm waterlogging disasters as shown in Table 3 according to the difficulty evaluation concept framework for residents’ evacuation at urban rainstorm waterlogging disasters and weighting method.

**Table 2:** Impact factor grading and scoring of evacuation behavior.

Factors Typ	Impact Factor Expert Score		
Level of education assignment	Junior middle school and below 1	High school and junior college 2	Bachelor degree or above 3
Types of profession assignment	Farmer	Worker	Student
	1	2	3
Evacuation channel type	Township roads 1	Provincial and municipal roads 2	Urban main roads 3
Optimal path choice assignment	Shortest path 1	Shortest time 2	Highest security 3
Shelter type assignment	Temporary shelter	District shelter	Municipal shelter
	1	2	3

Note: Table 2 graded and scored using expert scoring method.

**Table 3:** Difficulty evaluation index system and weight of residents’ evacuation at urban rainstorm waterlogging disasters.

Factor		Deputy Factor	Index	Weight
Waterlogging disaster Residents evacuation difficulty evaluation index	Dangerousness (H)	Rainfall factors	Precipitation greater than 50mm (X_H1_)	0.1692
		Waterlogging factor	Waterlogging depth (X_H2_)	0.2185
			Waterlogging flow velocity (XH_3_)	0.0329
	Road factors (E)	Road conditions	Road grade (X_E1_)	0.1365
			Road network density(X _E2_)	0.0683
	Residents’ own vulnerability (V)	Fragility of life is affected by water depth, flow velocity	0–14 years old, population of more than 60 years old (X_V1_)	0.1388
			Residents’ walking speed in water	0.1340
	Residents’ evacuation behavior (R)	Evacuation behavior characteristics	Types of profession(X_R1_)	0.0622
			Level of education(X_R2_)	0.1097
		Evacuation action selection	Quantity of shelter(X_R3_)	0.0374
			Evacuation method (X_R4_)	0.0206
			Evacuation channel type (X_R5_)	0.0440
			Shelter type (X_R6_)	0.0257

We conducted tests on weight calculation results by using AHP and the total ordering consistency ratio and the results are as follows: risk consistency ratio: 0.0025, weight to the overall target: 0.325; road factors consistency ratio: 0.0001, weight to the overall target: 0.2408; residents’ own vulnerability consistency ratio: 0.002, weight to the overall target: 0.1851; residents’ evacuation behavior consistency ratio: 0.0741, weight to the overall target: 0.2896.

### 6.3. Evaluation Model Building

The following difficulty index model of residents’ evacuation at urban rainstorm waterlogging disasters is established by using a weighted comprehensive evaluation method and an analytical hierarchy process, combined with the difficulty evaluation index system of residents’ evacuation during urban rainstorm waterlogging disasters:

(10) $$FBND=(H^{WH})(E^{WE})(V^{WV}){（1-R)}^{WR}$$

(11) $$H=W_{H1}X_{H1}+W_{H2}X_{H2}+W_{H3}X_{H3}$$

(12) $$E=W_{E1}X_{E1}+W_{E2}X_{E2}$$

(13) $$V=W_{V1}X_{V1}+W_{V2}X_{V2}\;$$

(14) $$R=W_{R1}X_{R1}+W_{R2}X_{R2}+W_{R3}X_{R3}+W_{R4}X_{R4}+W_{R5}X_{R5}+W_{R6}X_{R6}$$

where in the model, *FBND* is the difficulty index of residents’ evacuation at urban rainstorm water-logging disasters, which is used to express the level of difficulty of residents’ evacuation. The bigger the value, the more difficult the residents’ evacuation, and the easier on the contrary; the values of *H*, *E*, *V*, *R* represent the value of waterlogging disaster risk factors, road affecting factors, residents’ own vulnerability and residents’ own evacuation behavior influencing factors, respectively; whereas in the Equations (10–14), it is the value of index i after quantization, which is the weight of index *i*, representing the relative importance of the main factor of difficulty evaluation index of residents’ evacuation.

### 6.4. Results Analysis

The difficulty evaluation results are divided into three grades by using the difficulty evaluation index classification method of residents’ evacuation—quantile classification method and combing the actual situation of residents’ evacuation during rainstorm waterlogging disasters in Daoli District of Harbin: low difficulty (0–0.164), medium difficulty (0.164–0.355), high difficulty (0.355–0.564). In the evaluation results, the lower the evacuation difficulty evaluation, the easier of residents’ evacuation and the higher the evacuation difficulty evaluation, the more difficult the residents’ evacuation (Table 4 and Table 5).

**Table 4:** Difficulty evaluation results of residents’ evacuation under the scenario of a 5-year frequency rainstorm.

Item No.	Street Name	Road Waterlogging Length (m)	Evacuation Difficulty
1	Xinyang Road	80	Relatively difficult for evacuation
2	North Jianguo Liudao Street	60	Relatively difficult for evacuation
3	Central Avenue	30	Relatively difficult for evacuation
4	Diduan Street	50	Relatively difficult for evacuation
5	Intersection of Xinyang Road and Minqing Street	100	Difficult for evacuation
6	Zhaolin Street	120	Difficult for evacuation
7	Anfeng Street	100	Difficult for evacuation

**Table 5:** Difficulty evaluation results of residents’ evacuation under the scenario of a 100-year frequency rainstorm.

Item No.	Street Name	Road Waterlogging Length (m)	Evacuation Difficulty
1	Xinting Street	50	Relatively difficult for evacuation
2	Gongnong Avenue	60	Relatively difficult for evacuation
3	Ansheng Street	130	Relatively difficult for evacuation
4	Hesong Street	200	Relatively difficult for evacuation
5	Min’an Street	120	Relatively difficult for evacuation
6	Hexing Road	38	Relatively difficult for evacuation
7	Daoli South&North Road	55	Relatively difficult for evacuation
8	Weiwu Road	45	Relatively difficult for evacuation
9	Min’an Street	100	Relatively difficult for evacuation
10	Down Shitoudao Bridge	500	Difficult for evacuation
11	Intersection of Toulong Street and Maimai Street	300	Difficult for evacuation
12	Intersection of Toulong Street and Zhaolin Street	300	Difficult for evacuation
13	Intersection of Anfa Street and Jingwei 2nd Street	1500	Difficult for evacuation
14	Anguo Street	900	Difficult for evacuation

**Figure 8:**
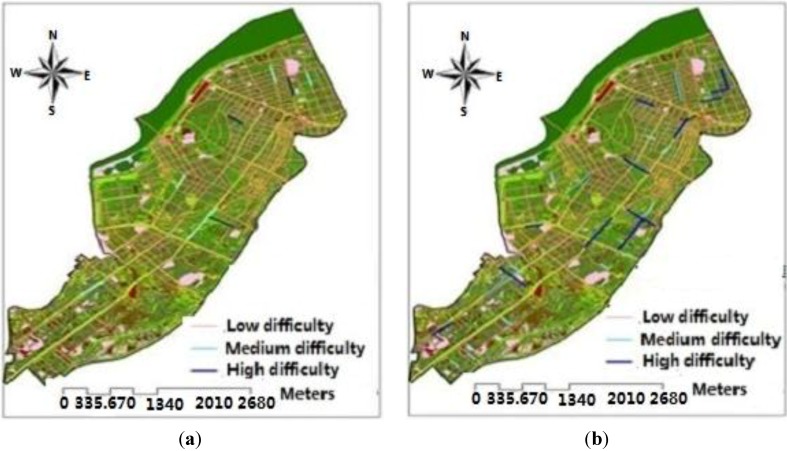
Difficulty evaluation of residents’ evacuation at urban rainstorm waterlogging disasters: (**a**) Difficulty evaluation of residents’ evacuation under the scene of 10-year; (**b**) Difficulty evaluation of residents’ evacuation under the scene of 50-year.

From Figure 8 we can see that, under the scenario of a 10-year frequency rainstorm, only certain road sections at three streets are classified as high difficulty grade, which indicates that residents’ evacuation is difficult for these road sections; and certain road sections in five streets are of medium difficulty grade which indicates that residents’ evacuation in these road sections is relatively difficult; the other areas are at low difficulty grade and residents’ evacuation is easier at this grade. Under the scenario of a 50-year frequency rainstorm, only certain road sections in five streets are of high difficulty grade, which indicates that residents’ evacuation in these road sections is difficult; and certain road sections in nine streets are of medium difficulty grade, which indicates that it is relatively difficult for residents to evacuate in these road sections; the other areas are at low difficulty grade and residents’ evacuation is easier at this grade. Under this scenario, only five streets cannot be used as evacuation routes during residents’ evacuation process.

## 7. Conclusions

This paper determined the influencing factors of residents’ evacuation through a questionnaire on residents’ evacuation behavior at urban rainstorm waterlogging disasters and residents’ walking in water experiments and selected the evaluation factors to establish an index system and model. The difficulty of residents’ evacuation at urban rainstorm waterlogging disasters has been divided into three levels. The difficult level of residents’ evacuation in each road in the research area has been evaluated according to the scenario simulation results and the results have been visualized by adopting GIS technology. The research result is the basis of preparing residents’ emergency shelters at urban rainstorm waterlogging disasters and evacuation studies, providing a decision basis to improve the level of urban disaster prevention and reduction and risk management, allowing residents to evacuate, their safe travel and so on.
